# Hydrothermal Synthesis of Nanostructured Manganese Oxide as Cathodic Catalyst in a Microbial Fuel Cell Fed with Leachate

**DOI:** 10.1155/2014/791672

**Published:** 2014-02-27

**Authors:** Yuan Haoran, Deng Lifang, Lu Tao, Chen Yong

**Affiliations:** ^1^Guangzhou Institute of Energy Conversion, Chinese Academy of Sciences, Guangzhou 510640, China; ^2^Key Laboratory of Renewable Energy and Gas Hydrate, Chinese Academy of Sciences, Guangzhou 510640, China; ^3^Guangzhou Division Academy, Chinese Academy of Sciences, Guangzhou 510070, China

## Abstract

Much effort has been devoted to the synthesis of novel nanostructured MnO_2_ materials because of their unique properties and potential applications as cathode catalyst in Microbial fuel cell. Hybrid MnO_2_ nanostructures were fabricated by a simple hydrothermal method in this study. Their crystal structures, morphology, and electrochemical characters were carried out by FESEM, N_2_-adsorption-desorption, and CV, indicating that the hydrothermally synthesized MnO_2_ (HSM) was structured by nanorods of high aspect ratio and multivalve nanoflowers and more positive than the naturally synthesized MnO_2 _(NSM), accompanied by a noticeable increase in oxygen reduction peak current. When the HSM was employed as the cathode catalyst in air-cathode MFC which fed with leachate, a maximum power density of 119.07 mW/m^2^ was delivered, 64.68% higher than that with the NSM as cathode catalyst. Furthermore, the HSM via a 4-e pathway, but the NSM via a 2-e pathway in alkaline solution, and as 4-e pathway is a more efficient oxygen reduction reaction, the HSM was more positive than NSM. Our study provides useful information on facile preparation of cost-effective cathodic catalyst in air-cathode MFC for wastewater treatment.

## 1. Introduction

Microbial fuel cell (MFC) is a promising biotechnology that utilizes microorganisms as catalysts to decompose organic or inorganic matter and simultaneously harvest electricity, which present a new approach for generating electricity from waste and biomass [1–4]. Air breathing microbial fuel cells, typically characterized by using natural convection air-flow to their cathodes, are attractive for wastewater treatment applications due to their simple single-chamber construction and their unique ability to remove organic matter and generate bioelectricity. In such oxygen cathode system, the function of MFC would be significantly affected by the cathode performance due to the poor kinetics of oxygen reduction reaction (ORR).

To improve ORR and simultaneously maximize the power density, various kinds of electrocatalysts such as Pt [5], lead dioxide [6], iron (III) phthalocyanine(FePc) and cobalt tetramethoxyphenyl porphyrin(COTMPP) [7–10], Prussian blue/polyaniline [11], iron related ethylenediaminetetraacetic acid [12], Co/Fe/N/CNT [13], and Co-naphthalocyanine [14] have been evaluated for their ORR activity in MFC cathodes and the MFCs all exhibited good performances. However, the high cost of platinum, the toxicity of lead dioxide, long-term instability of the transition metal macrocycles, and phthalocyanines make these alternatives impractical.

In the past decade, manganese dioxide has been studied as one of the most promising cathode catalyst for alkaline fuel cell and metal-air batteries application [15], and several research groups have previously shown that nonprecious manganese dioxide electrocatalysts were highly efficient for catalyzing ORR and lowering overall cost at the same time [16–18]. Recent studies had paid their attention towards carbon nanotubes (CNTs) coated with manganese dioxide and MnO_2_ nanoparticles [19–21]. However, very limited efforts have been made to evaluate the activities of nanostructured manganese oxides. Roche et al. [22] found that the power density of the MFC with carbon-supported MnO_2_ nanoparticles can reach 161 mW/m^2^ compared to 19 mW/m^2^ for a benchmark Pt/C at room temperature, and when using nanostructured MnO_*x*_ as cathode catalyst the MFC can achieved a peak power density of 772.8 mW/m^3^ [23]. Thus, it is of significant to develop manganese oxides with controllable morphology and nanostructures by using facile methods for enhancing oxygen reduction and lowering the cost of single-chamber MFC for wastewater treatment. In this study, nanostructure manganese oxide prepared by hydrothermal synthesis method is applied on MFC cathode, which shows comparable catalytic capability to naturally synthesized manganese oxide, and it could further facilitate the scaling up of MFC.

## 2. Materials and Methods

### 2.1. Catalysts Preparation

The hydrothermally synthesized nanostructure MnO_2_ (HSM) was synthesized as described elsewhere [24]. Briefly, 0.2 g MnSO_4_·H_2_O and 0.5 g KMnO_4_ were dissolved in 100 mL distilled water; then well-mixed aqueous solution of KMnO_4_ and hydrated MnSO_4_ were transferred to a Teflon-lined pressure vessel (QiangQiang Instrument, Shanghai.) and loaded into an oven preheated to 140°C; the dwell time for the reaction chose 8 h when the nanostructure MnO_2_ was prepared. After the reaction was finished, the pressure vessel cooled to room temperature naturally. The precipitation formed was filtered and washed with distilled water until the pH of the wash water was 7 and finally dried at 100°C in air. The same amounts of starting materials were left in a beaker overnight for the formation of MnO_2_ precipitate in order to see the structural evolution of nanostructured MnO_2_ from room temperature to the hydrothermal treatment. After the reaction was finished, all operations were the same as the synthesis of nanostructure MnO_2_.

### 2.2. Characterization of MnO_**2**_

#### 2.2.1. FESEM

The morphology of MnO_2_ was characterized with field emission scanning electronic microscopy (FESEM) (HITACHI, S-4800). The specific surface areas of MnO_2_ were measured by the Brunauer-Emmett-Teller (BET) method, in which N_2_ adsorption at 77 K was applied and Carlo Erba Sorptometer was used.

#### 2.2.2. CV

Cyclic voltammetric (CV) measurements were performed with an Autolab potentiostat (model PGSTAT 30) with a three-electrode system (Ecochemie, Netherlands). The glass carbon electrode (GCE, with a diameter of 3.0 mm) coated by catalyst severs as the working electrode a Pt wire and Ag/AgCl (sat. KCl, 222 mV versus SHE) were used as the counter and reference electrodes, respectively. CV measurements were performed from –0.6 V to 0.2 V at a scan rate of 100 mV·S^−1^ in a 0.l M KOH electrolyte. The electrolyte solution is bubbled with O_2_ or N_2_ to establish aerobic or anaerobic environment, respectively, for 30 min prior to each scan series, and 3 min between every two scans.

### 2.3. MFC Configuration and Operation

All MFCs were operated at 30 ± 1°C in a temperature-controlled incubator (HPG-280H, China). The air-cathode MFC consisted of a plastic (Plexiglas) cuboid chamber (2 × 5 × 5 cm^3^) [25] and with a membrane electrode assembly on one side. Carbon felt (8 × 8 cm^2^, Panex 33160K, Zoltex) was used as the anode. Carbon cloth and cation-exchange membrane was hot-pressed together to be cathode. Titanium wire was inserted inside the carbon felt and carbon cloth to connect the circuit. Active area of the cathode was 25 cm^2^. For all tests, a 1000 Ω external resistance was fixed except as noted. And the anode chamber of the MFC was filled with 40 mL of leachate (collected during the biodrying pretreatment of MSW from Boluo waste treatment).

### 2.4. Data Acquisition

The cell voltage outputs were measured by a precision multimeter (Victory 9807A, China) and a 16-channel voltage collection instrument (AD8223, China) which continuously monitored the voltages and transferred data to the computer at an interval of 2 min. To obtain a polarization curve, the external resistor varied from 50 Ω to 10000 Ω when the voltage output approached steady state. The corresponding voltages at different external resistances were recorded and the power output (W), power density (W·m^−2^), and current output density (A·m^−2^) were calculated according to *P* = *U* ^2^/*R*, *P* = *IU*/*A*, *I* = *U*/*RA*, where *U*(V) is the measured voltage, *I*(A) is the current, *R*(Ω) is the external resistance, and *A*(cm^2^) is the active surface area of the cathode, and individual electrode potentials were measured versus saturated calomel electrode (SCE). The external resistance was fixed at 1000 Ω throughout all the experiments except as noted.

## 3. Results and Discussion

### 3.1. Synthesis and Characterization of the Catalysts

**Figure 1:**
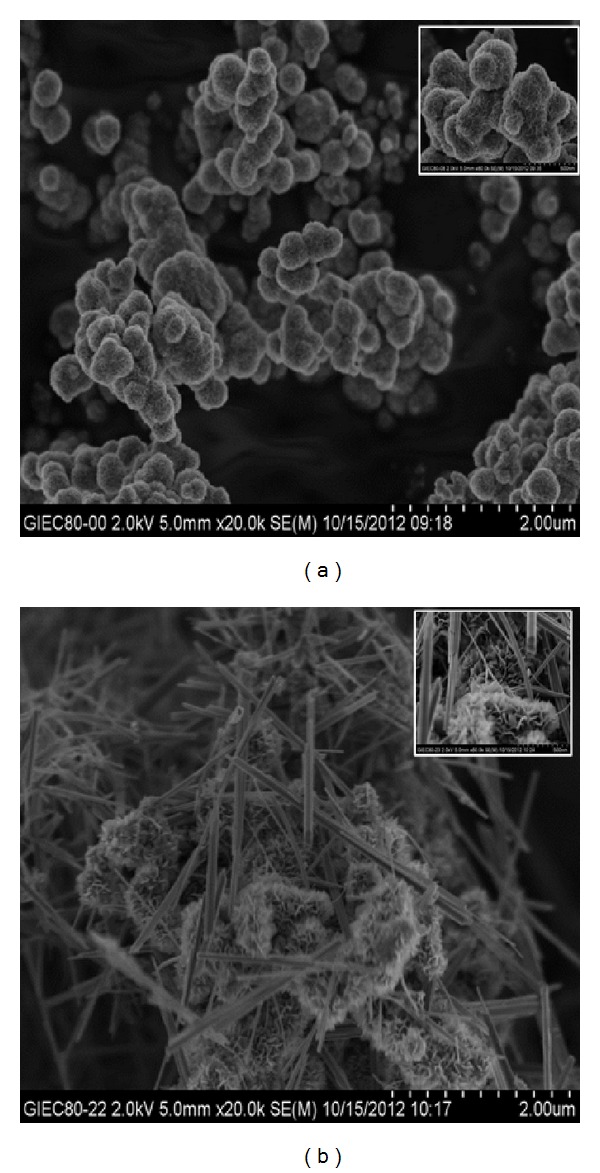
SEM images of MnO_2_ prepared by different methods: (a) natural process; (b) hydrothermal process. The inset images are the higher magnifications.

The nanostructured MnO_2_ is synthesized by hydrothermal method and MnO_2_ (NSM) is precipitated at room temperature naturally. SEM images of the obtained MnO_2_ are displayed in Figure 1. As can be seen from the pictures, the flowerlike whiskers of MnO_2_ were formed as the material prepared at room temperature by natural synthesis, and when hydrothermally treated for 8 h, there has been an increase in the size of the individual whiskers which replicates the formation of nanostructured surface with a distinct platelike morphology, and the nanoarchitecture with few rods evolving in addition to nanostructured platelike morphology was observed, the same as Subramanian found [24]. Moreover, the BET surface areas of hydrothermal and NSM were determined to be 24.91 and 111.89 m^2^/g, respectively (Table 1). For the HSM, the nanostructure increases the BET surface area and is easier for the organic substrates to be adsorbed on the cathodes, and the high BET surface areas of MnO_2_ catalysts could enhance the oxygen absorption and electron acceptance on catalysts surface. Oxygen vacancies created to fulfill an overall charger balance can migrate onto the surface of MnO_2_ nanorod and play important roles in catalysis [18]. With the nanorod surface properties and the existence of oxygen vacancies, the MnO_2_ should substantially increase the oxygen reaction rate and electron acceptance capability. On the other hand, the specific nanorod and platelike structure of the MnO_2_ catalysts facilitated oxygen adhesion.

### 3.2. Electrochemical Characterization of the Catalysts

**Figure 2:**
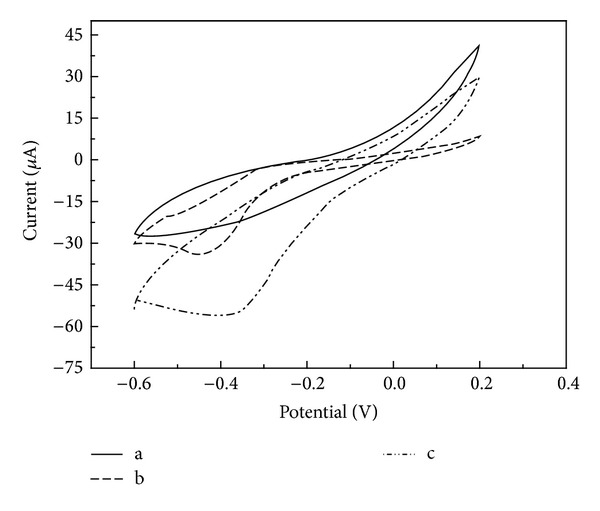
Cyclic voltammograms of MnO_2_ for ORR at scan rate of 100 mV/s in 0.l M KOH. (a) Electrolyte bubbled with N_2_; (b) NSM; (c) HSM. (b)-(c) Electrolyte bubbled with O_2_.

Cyclic voltammograms recorded at scan rate of 100 mV/s for naturally and hydrothermally synthesized MnO_2_ in a 0.l M KOH electrolyte under aerobic (bubbled with O_2_) and anaerobic (bubbled with N_2_) environment are shown in Figure 2. It can be seen from the figures that the MnO_2_ possesses a reduction peak (−0.5 to −0.3 V) in aerobic environment but no peak in anaerobic environment (Figure 2), indicating the peak attribution to the catalyzed ORR process.

Comparing with the naturally synthesized MnO_2_, the peak potential of HSM was −0.385 V versus Ag/AgCl, more positive than that for naturally synthesized MnO_2_ (−0.443 V) and the HSM with a noticeable increase in oxygen reduction peak current (Figure 2). This may suggest an effective disproportionation of the electrogenerated hydrogen peroxide by the HSM nanorods and nanostructured platelike [23]. More importantly, the improved dispersion of nanostructured MnO_2_ favors oxygen adsorption due to its larger BET, facilitating electron transfer through the film and decreasing the ORR over potential. In addition, the presence of oxygenated groups on the surface of cathode catalyst, partially due to oxidation by permanganate, may facilitate oxygen reduction, as reported by Kinoshita [26]. Thus, as the NSM is relatively low adsorption of oxygen and weaker ORR performance, it was expected that between the naturally and hydrothermally synthesized MnO_2_, the HSM would constitute a more effective cathode catalyst material for MFCs.

### 3.3. MFC Performances with Various Catalysts

**Figure 3:**
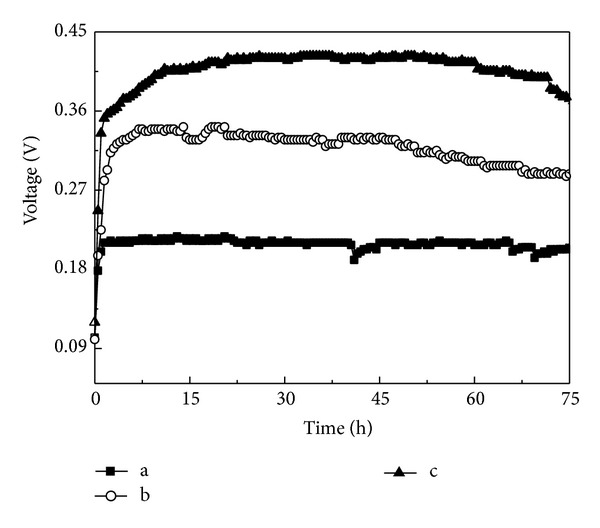
The voltage of MFCs with different cathode catalysts. (a) Cathode without loading catalyst; (b) cathode loading with NSM; (c) cathode loading with HSM.

The performance of MFCs with the NSM and HSM was evaluated alongside that of the cathode without loading catalyst by monitoring cell voltage output, anode and cathode polarization, and power density. As shown in Figure 3, a maximum stable voltage of 0.42 V was delivered by the MFC loading with HSM, which was larger than loading with NSM (0.34 V) and without loading catalyst (0.21 V) MFCs achieved. The main reason for the higher power generation of HSM MFC was that HSM possessed high oxygen reduction rates (ORRs), and the low voltage generation of MFC without loading catalyst could be explained by its higher *R*_in_ (250 Ω, as shown in Table 1). The difference of *R*_in_ among these MFCs may have been due to the electrical characteristics of the various catalysts, particularly conductivity [18]. Therefore, the sufficient dispersion of nanostructured MnO_2_ over the cathode surface resulted in high conductivity and decreased the cathodic resistance, thus achieving a better performance in the MFC loading with HSM.

**Figure 4:**
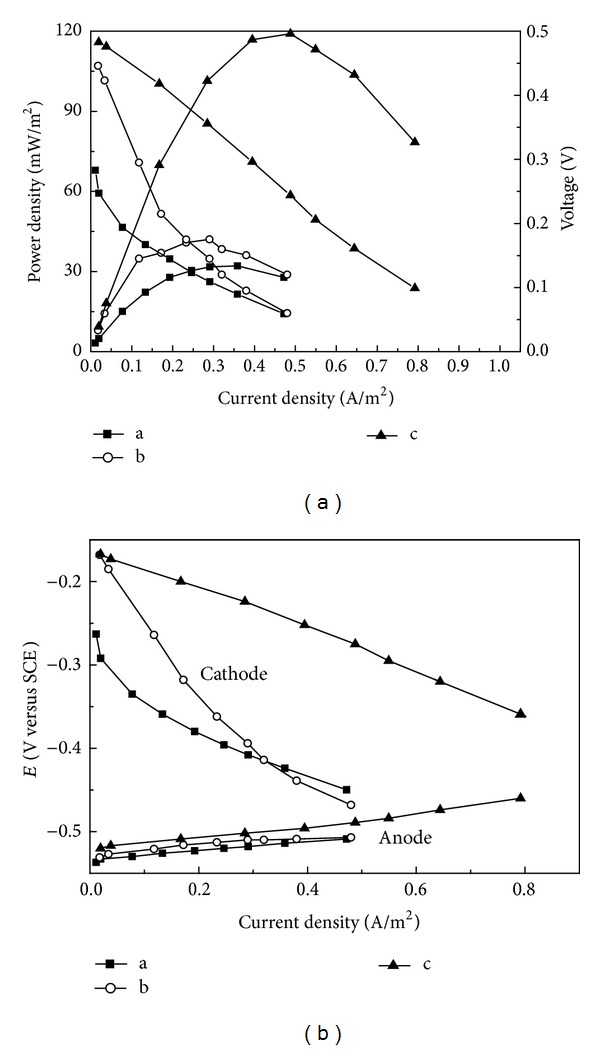
Performance of MFC equipped with different catalysts. (a) Cathode without loading catalyst; (b) cathode loading with NSM; (c) cathode loading with HSM.

Power densities of different catalysts were compared using a polarization curve measurement. The HSM-based MFC had the highest maximum power density than NSM-based MFC and without loading catalyst MFC. The Maximum power density (based on 25 cm^2^ projected anode surface area) of 119.07 mW/m^2^ (about 5.95 W/m^3^ based on 50 cm^3^ anode volume, which was about 6.7 times higher than electrochemically deposition nanostructured MnO_*x*_ (772.8 mW/m^3^) [23]), was obtained with HSM as cathode catalyst (with a current density of 0.49 A/m^2^), while a maximum power density of 32.11 mW/m^2^ and 42.05 mW/m^2^ was achieved at current densities of 0.22 A/m^2^ and 0.29 A/m^2^ without any catalyst or with NSM as cathode catalyst (Figure 4(a)), about 73.03% and 64.68% lower than that with HSM, respectively. The results of this investigation on the dependency of power generation in MFCs are consistent with the results of the BET studies. To understand this observation better, the individual electrode potentials were also measured (Figure 4(b)). The anodic potentials were almost identical in all case due to the matured anodic biofilms, whereas the cathodic potentials were varied, so it was evident that the cathode was the limiting factor in these MFC reactors. For instance, in hydrothermally synthesized MnO_2_ MFC, with the increased current densities of 0–0.8 A/m^2^, the anode potential increased insignificantly from −0.52 to −0.46 V, whereas the cathode potential dropped from −0.17 to −0.36 V; the larger driving force with an over potential of 0.19 V required for the cathode compared to the value of 0.06 V required for the anode indicates that power generation from the MFC was dominated by cathode polarization. This is also consistent with the higher OCV (Table 1) and the lower internal resistance of HSM-based MFC than NSM-based MFC, since the lower internal resistance would result in a less ohmic loss and less polarization [27]. Therefore, the results indicated that MnO_2_ prepared with a hydrothermal synthesis method could be effectively used as a catalyst in single-chamber air-cathode MFCs to generate current.

### 3.4. Oxygen Reduction Mechanisms

The ORR mechanism in alkaline media on MnO_*x*_ is usually described by the partial 2-electron reduction of O_2_ as follows:

(1) $$\begin{array}{c} {\text{O}}_{2}+{\text{H}}_{2}\text{O}+2{\text{e}}^{-}⟶\text{H}{{\text{O}}_{2}^{}}^{-}+{\text{OH}}^{-} \end{array}$$

(2) $$\begin{array}{c} {{\text{HO}}_{2}^{}}^{-}+{\text{H}}_{2}\text{O}+2{\text{e}}^{-}⟶3{\text{OH}}^{-} \end{array}$$

(3) $$\begin{array}{c} 2{{\text{HO}}_{2}^{}}^{-}⟶{\text{O}}_{2}+2{\text{OH}}^{-} \end{array}$$

Manganese oxides were found to facilitate the decomposition of hydrogen peroxides, according to the HO_2_^−^ disproportionation reaction (3) [28]. Oxygen can then be reduced according to the reaction (1); the overall reaction is then the apparent 4-electron reduction of O_2_:

(4) $$\begin{array}{c} {\text{O}}_{2}+2{\text{H}}_{2}\text{O}+4{\text{e}}^{-}⟶4{\text{OH}}^{-} \end{array}$$

The 4-electron process to combine oxygen with electrons and protons directly to produce water as the end product; however, 2-electron processes involving the information of hydrogen peroxide ions as the intermediate. And the hydrogen peroxide ions are corrosive and can degrade the membrane and/or corrode the fuel cell cathode [29, 30]. Furthermore, Cao et al. [31] have studied the mechanism of the ORR in several MnO_2_-catalysed air electrodes. It was found that the ORR is accompanied by the reduction of MnO_2_ and that the catalytic activity is dependent on the electrochemical redox activity of these species. In addition, the oxygen reduction at MnO_2_-catalyzed air cathode proceeds through chemical oxidation of the surface Mn^3+^ ions generated by the discharge of MnO_2_ rather than through a direct two-electron reduction as previously suggested.

According to Bard and Faulkner [32], the number of electron transfer (*n*) involved in the oxygen reduction at 25°C could be estimated with Randles-sevcik equation (5):

(5) $$\begin{array}{c} i_{p}=0.4463{\left(Fn\right)}^{\left(2/3\right)}{AD}_{o}C_{o}^{*}V^{\left(1/2\right)}{\left(RT\right)}^{\left(-1/2\right)}, \end{array}$$

where *i*_*p*_ is peak current (A), *F* is Faraday's constant (96485 C/mol), *n* is the number of electrons appearing in half-reaction for the redox couple, *A* is the electrode area (cm^2^), *D*_*o*_ is the electrolyte's diffusion coefficient (cm^2^/s), *C*_o_* is the concentration of electrolyte at the electrode surface (mol/cm^3^), *V* is the potential scanning rate (v/s), *R* is the universal gas constant (8.314 J/mol·K), and *T* is the absolute temperature (*t* + 273.15 K). At 25°C, for *A* in cm^2^, *D*_*o*_ in cm^2^/s, *C*_o_* in mol/cm^3^, and *V* in v/s, *i*_*p*_ in amperes is as follows:

(6) $$\begin{array}{c} i_{p}=\left(2.69\times 10^{5}\right)n^{\left(2/3\right)}{AD}_{o}C_{o}^{*}V^{\left(1/2\right)}. \end{array}$$

*A* in this study was approximately 0.07 cm^2^. Then according to the data shown in Figure 2, *n* in NSM is calculated to be 2.2, but in HSM it is calculated to be 4.3, suggesting that an apparent 2-e reduction of oxygen was achieved on the NSM in alkaline pH solution, but an apparent 4-e reduction of oxygen was achieved on the HSM. This result is the same as the one on MnO_*x*_/C using ring-disc electrode in alkaline solution [22] and the one on electrochemically deposition MnO_*x*_ nanorods in neutral solution [23]. Moreover, the previous studies indicated that 4-e pathway is more efficient than 2-e pathway [30, 33]. This result further confirmed the previous outcome in this study.

## 4. Conclusions

In this study, by hydrothermal synthesis method a nanorods evolving in addition to nanostructure platelike morphology MnO_2_ is synthesized, characterized, investigated by SEM and CV methods in alkaline solution and finally incorporated into air-cathode MFCs as cathode ORR catalysts. It is shown that the nanostructure MnO_2_ has quite good capability for ORR catalysis and has an electrochemical activity towards ORR via a 4-e pathway in alkaline solution which is more efficient than 2-e pathway as the NSM undergo. When the MnO_2_ are applied onto air-cathode MFC, the performance of the nanostructured MnO_2_-based MFC is more efficient and stable than the natural synthesis MnO_2_. Our findings provide useful information to develop appropriate nanostructured MnO_2_ catalysts towards oxygen reduction in MFC using this facile method. Due to its low cost, easy preparation, and good MFC performance, this catalyst could be a very promising electrocatalyst for air-cathode MFC. It is believed that this efficient and economic catalyst could facilitate the scaling up and commercialization of MFC reactors for practical applications.

## Figures and Tables

**Table 1:** Performance of MFCs based on different cathodic catalysts.

Catalyst	OCV (V)	Internal resistance (Ω)	Maximum power density (mW/m^2^)	Maximum current density (A/m^2^)	BET (m^2^/g)
Without catalyst	0.33	250	32.11	0.22	—
Naturally synthesized MnO_2_	0.47	200	42.05	0.29	24.91
Hydrothermally synthesized MnO_2_	0.50	150	119.07	0.49	111.89
